# Assessing the presence, concentrations, and potential ecological impacts of trace metal contamination in the Potomac River Test Range Complex middle danger zone

**DOI:** 10.1007/s10661-026-15382-2

**Published:** 2026-05-01

**Authors:** Tyler Frankel, Stephen Hanna, Leanna Giancarlo

**Affiliations:** 1 Department of Earth and Environmental Sciences, University of Mary Washington, 1301 College Ave, Fredericksburg, VA 22401 USA; 2 Department of Geography, University of Mary Washington, 1301 College Ave, Fredericksburg, VA 22401 USA; 3 Department of Chemistry and Physics, University of Mary Washington, 1301 College Ave, Fredericksburg, VA 22401 USA

**Keywords:** Underwater military munitions, Environmental indices, Metals

## Abstract

This study evaluated the presence, spatial distribution, and ecological risk of trace metal contamination in the Potomac River Test Range Complex (PRTR) middle danger zone, a heavily utilized military testing site with over a century of munitions activity. Surface water, near-bed water, and surficial sediment samples were collected from 21 sites and analyzed for trace metals (Al, As, B, Cd, Co, Cr, Cu, Fe, Mg, Mn, Ni, Pb, and Zn) using inductively coupled plasma optical emission spectroscopy. Contamination levels were interpreted using the Heavy Metal Pollution Index (HPI) for water samples and the Geoaccumulation Index (Igeo) and Potential Ecological Risk Index (PERI) for sediments. Ward’s multivariate cluster analysis with silhouette optimization was applied to assess spatial relationships between contamination patterns and historical projectile-density zones. Surface waters were generally low in contamination, whereas near-bed waters exhibited higher metal concentrations with multiple sites classified as critically polluted. Sediments showed widespread anthropogenic enrichment (Igeo Classes 1–3), with elevated ecological risks (PERI) concentrated in areas of dense historical munitions activity. Cluster analyses revealed clear spatial separation between high- and low-intensity use zones, supporting a linkage between munitions testing and localized contamination. These findings demonstrate that military activities contribute measurably to metal contamination superimposed on broader watershed inputs. This study represents one of the first field-based, multi-matrix assessments of contamination within the PRTR, advancing beyond prior evaluations that utilized modeling and screening approaches. This integrated framework provides improved resolution of contamination pathways and strengthens targeted monitoring, ecological risk assessment, and management of impacted aquatic systems.

**Supplementary Information:**

The online version contains supplementary material available at 10.1007/s10661-026-15382-2.

## Introduction

Aquatic ecosystems have been utilized for military training exercises and weapons testing due to their advantages in human safety, logistics, and performance characterization. However, these activities have resulted in the accumulation of underwater military munitions (UWMM) and associated contaminants in many aquatic environments. Many waterways have also been contaminated by the historic dumping of waste munitions which was banned in the United States by the Marine Protection, Research, and Sanctuaries Act of 1972 (Congress, 1972). Used or discarded UWMM contain a variety of organic and inorganic constituents, including 2,4,6-trinitrotoluene, 1,3,5-trinitro-1,3,5-triazine, octahydro-1,3,5,7-tetranitro-1,3,5,7-tetrazocin, lead (Pb), zinc (Zn), copper (Cu), chromium (Cr), manganese (Mn), mercury (Hg), cadmium (Cd), nickel (Ni), antimony (Sb), vanadium (V), and cobalt (Co) (Shukla et al., 2023). Many of these have been shown to bioaccumulate in organisms (Koide et al., 2016), cause genotoxic, cytotoxic, and carcinogenic effects on aquatic wildlife (Lotufo, 2017), and pose risks to human health through accidental exposure during occupational (Beck et al., 2019) or recreational activities (Francis, 2011). Contaminants can enter surrounding environments through deposition of propellant and primer residues during firing and detonation (Hewitt et al., 2005), release from partially exploded or breached munitions (Beck et al., 2019), and long-term corrosion of outer casings after submersion (Li et al., 2016). Due to varied hydrodynamic conditions, physiochemical processes, and degradation pathways, contaminant dissolution and release rates can fluctuate substantially over time (Beck et al., 2019). Although corrosion has been shown to be inhibited by sediment encapsulation and anoxic conditions (George et al., 2015), the extent and progression of corrosion at legacy sites are poorly understood (Beddington & Kinloch, 2005). Recent studies have also shown that trace metal mobility and persistence in aquatic ecosystems are strongly controlled or influenced by hydrochemical conditions and sediment–water interactions (Benmarce et al., 2024; Gori et al., 2023). Additionally, there is significant uncertainty regarding the exact locations of UWMM due to poor or non-existent recordkeeping (Brewer & Nakayama, 2008). Elevated concentrations of munition-associated contaminants have been detected in sediments adjacent to partially or fully buried UWMM in multiple environments (Beck et al., 2025; Briggs et al., 2016). While studies examining UWMM impacts have primarily focused on marine environments due to the impacts of relic munitions resulting from the two World Wars (WWI and WWII) (Beck et al., 2018), brackish and freshwater ecosystems are also at increased risk and remain relatively understudied (Francis, 2011).

**Fig. 1:**
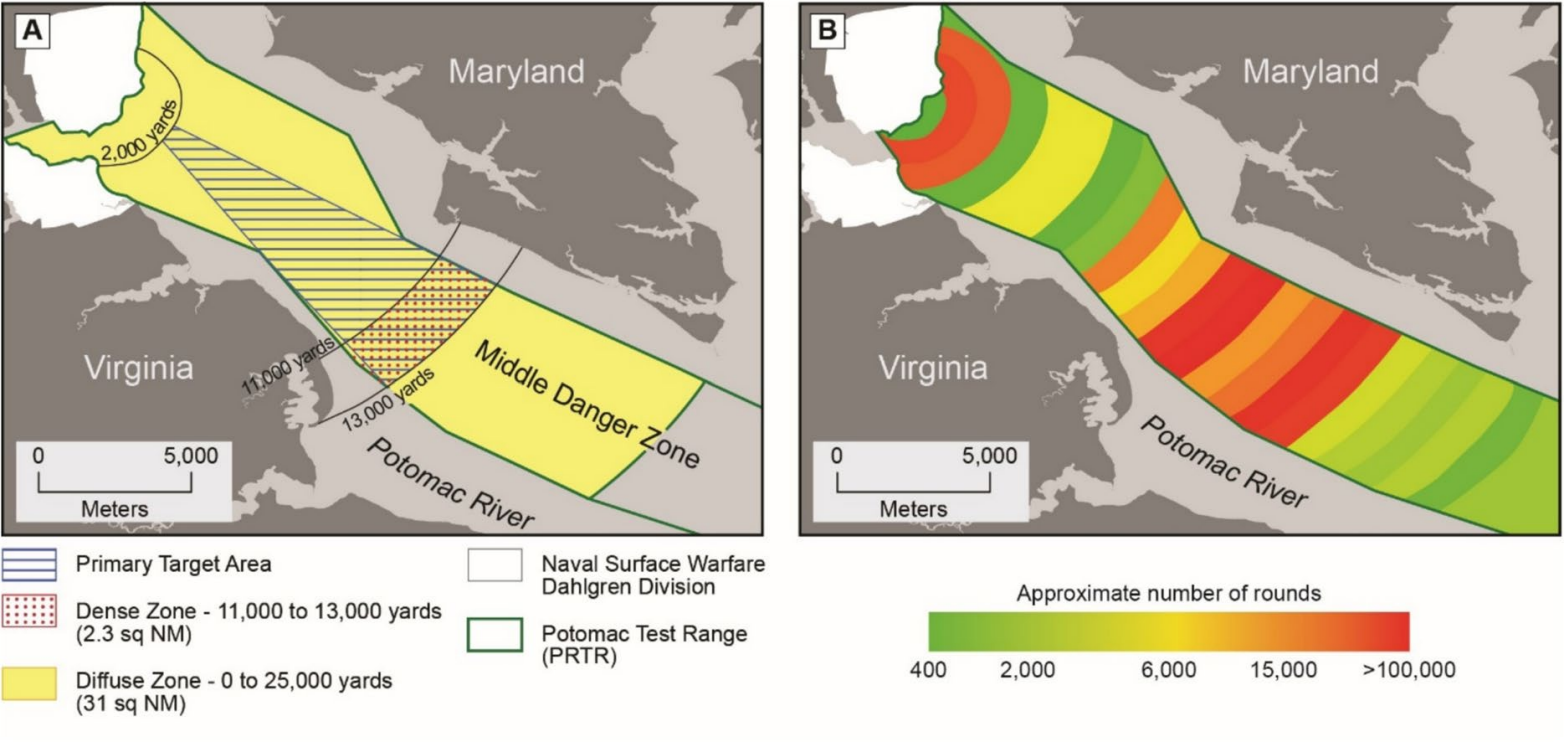
Maps identifying **A** the primary target, diffuse, and dense zones of the middle danger zone and **B** the estimated distribution of large-caliber projectiles in the middle and lower danger zones. From: Naval Surface Warfare Center (2013)

As the second largest watershed within the Chesapeake Bay, the Potomac River extends over 405 miles in length and flows through regions of Virginia, West Virginia, Pennsylvania, Maryland, and the District of Columbia. With an estimated basin population of roughly 6.89 million people, the river provides important provisioning, regulating, and cultural services (Interstate Commission on the Potomac River Basin, 2015). Consequently, it has been impacted by multiple anthropogenic contamination sources including agricultural and urban runoff, wastewater effluent, combined sewage overflows, and industrial pollution (Ator, 1998; Sapp et al., 2024). Established in 1918 by the US Navy, the Potomac River Test Range (PRTR) Complex is the largest fully instrumented, over-water gun firing range and munitions testing center in the United States. Spanning 51 nautical miles of the Potomac River, the range is divided into three distinct geographic zones (upper, middle, and lower zone), with the middle zone receiving the heaviest historical and current use (Naval Surface Warfare Center 2013) (Fig. 1).

These zones are open for recreational or commercial activities when not actively being utilized for testing, creating the potential for unintended public interaction with UWMM and associated contaminants. Despite over a century of operations, relatively few peer-reviewed studies have been conducted to understand and identify the potential impact of testing activity on the river and populations that utilize it. While much of the information on historical ordinance use is based on anecdotal sources, estimates of the number of large-caliber projectiles fired into each danger zone between 1918 and 2007 and the total weight of chemical constituents have been provided as part of a water range sustainability environmental program assessment (Naval Surface Warfare Center 2013). Although this assessment concluded that the concentrations of munitions constituents were orders of magnitude below levels expected to cause adverse effects to human health or the environment, several limitations were acknowledged. No direct field-based water or sediment field sampling was performed as part of this study and conclusions were drawn exclusively from fate/transport modeling and the application of a screening network. Munition-use inputs were incomplete and, in some cases, reconstructed using averages from adjacent time periods and assumptions of comparable activity. Finally, key datasets utilized for model parameterization and comparison range from 1972 to 2008 (Glenn, 1988; Goodwin et al., 1984; Houser & Fauth, 1972; United States Environmental Protection Agency (USEPA) 2002; Versar, 2008) may not adequately reflect current sediment conditions or the release of contaminants from older compromised UWMM.

Based on the above information, several critical knowledge gaps exist including (1) the absence of field-based measurements of trace metals in various mediums within the PRTR middle danger zone, (2) a limited understanding of spatial contamination patterns relative to projectile distributions, and (3) a lack of integrated ecological risk assessments to evaluate potential impacts to humans and wildlife. Thus, this study was designed to assess the presence and concentrations of trace metal contamination in the middle danger zone of the Potomac River and to evaluate whether spatial patterns are consistent with historical and ongoing munitions-testing activity in the PRTR. Based on the primary munitions constituents listed in Table 1 (Naval Surface Warfare Center 2013) the decision was made to focus on trace metal contamination.

**Table 1:** Estimated trace metal constituents in live and inert projectiles fired on the PRTR from 1918 to 2007 by total weight adapted from Naval Surface Warfare Center (2013)

Constituent	Total sum of weight (lbs)
Iron	30,980,921.82
Copper	958,087.21
Manganese	463,238.57
Aluminum	148,631.69
Zinc	61,467.90
Nickel	47,957.43
Lead	8,417.13
Zinc phosphate	1,777.80
Chromium	442.15
Cadmium	186.94
Lead naphthenate 36%	103.52
Magnesium powder	77.08
Lead azide	55.43
Zinc chromate	37.55
Lead styphnate	16.27

Non-essential elements such as arsenic (As), Cd, and Pb are persistent (Edo et al., 2024), exhibit toxic effects at relatively low concentrations (Jomova et al., 2025; Naz et al., 2023), bioaccumulate in aquatic organisms (Naz et al., 2023; Sartorius et al., 2024), and are associated with various diseases and cancers in humans (Luo et al., 2011; Tshala-Katumbay et al., 2015; Wang et al., 2011). Because metal contamination can arise from both geogenic and anthropogenic sources, multiple complementary indices have been developed to distinguish anthropogenic contributions of metals in water and sediments and assess risk to wildlife and humans. The Heavy Metal Pollution Index (HPI) evaluates water quality relative to regulatory thresholds (Singh et al., 2024), the Geoaccumulation Index (Igeo) assesses sediment contamination relative to background concentrations (Müller, 1969), and the Potential Ecological Risk Index (PERI) incorporates both concentration and toxicity to evaluate ecological risk (Hakanson, 1980). These indices are widely applied in contemporary environmental geochemistry studies to evaluate contamination and risk across aquatic systems (Barberio et al., 2020; Gori et al., 2023; Sappa et al., 2020), providing a robust framework for interpreting complex contamination patterns.

This study provides the first field-based, multi-matrix assessment of trace metal contamination in the PRTR middle danger zone and represents one of the first efforts to establish a causal link between observed contamination patterns with munitions-use intensity in an active military testing environment. The remainder of this paper describes the study area and methods, presents analytical procedures and index calculations, reports results, discusses findings in the context of other contaminated aquatic systems, and provides conclusions and management implications.

## Methods

### Site description

**Fig. 2:**
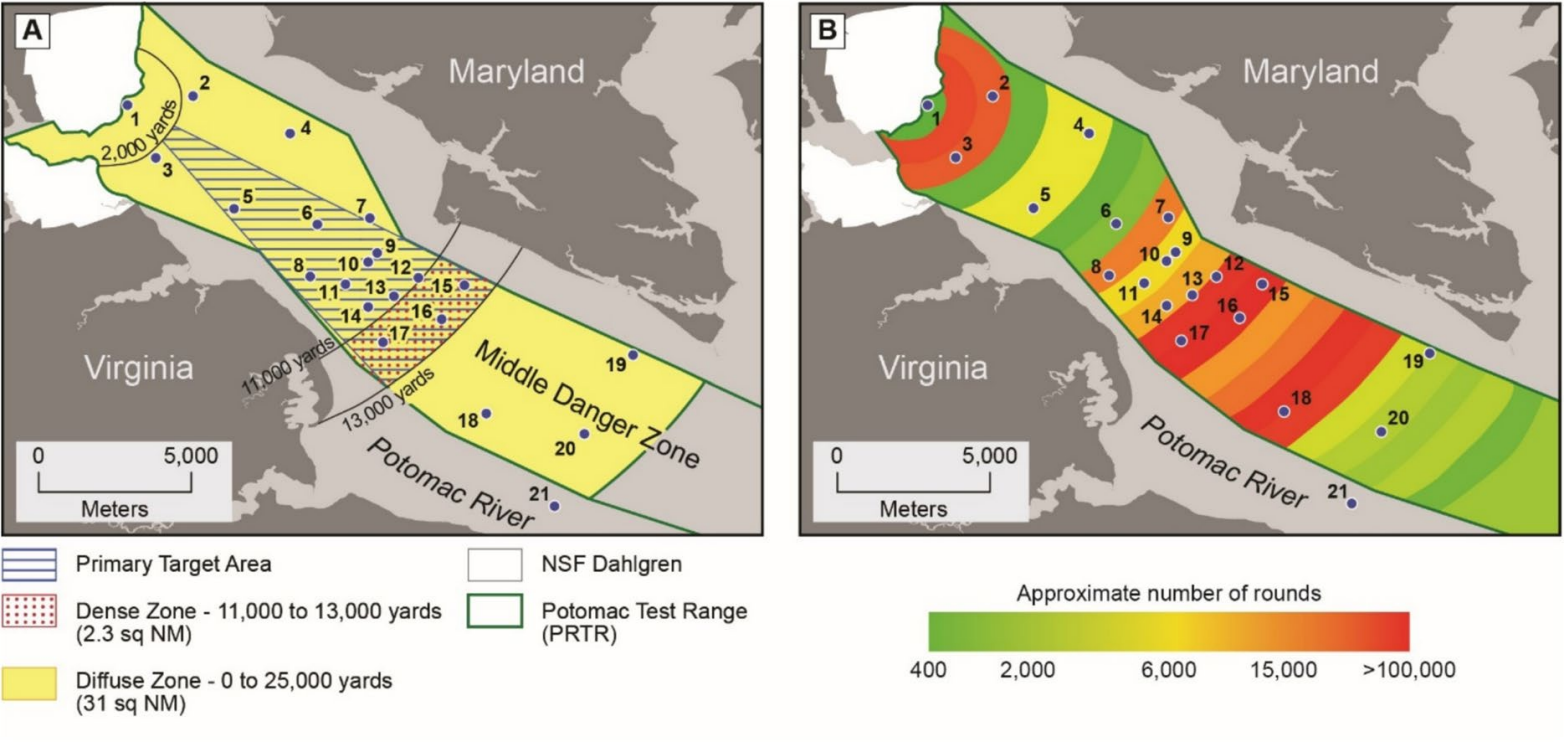
Map identifying locations where surface water, near-bed water, and sediment samples were obtained relative to the diffuse, dense, and primary target areas (**A**) and approximate number of large-caliber projectiles fired (**B**). Numbers indicate sampling location IDs. Figure adapted from Naval Surface Warfare Center (2013)

Sampling was conducted on June 1, 2025, within the PRTR middle danger zone which extends from the Dahlgren Naval Base downstream to St. Clement’s Island. Sampling sites were selected to represent locations upstream, adjacent, and downstream of the heaviest distribution of large-caliber projectiles based on information provided by the Naval Surface Warfare Center (2013) (Fig. 2a and b). Surface water, near-bed water (6 inches above the channel bed), and sediment samples were collected from 21 different locations as shown in Fig. 2 and analyzed using the methods described below. Samples from site 21 were collected outside of the diffuse zone due to accidental tidal drift during sampling. One near-bed water sample (site 1) was lost during transport.

### Trace metal quantification

Surface water samples were collected in precleaned, acid-washed 200-mL polyethylene bottles. Bottles were rinsed three times in river water before the final sample was obtained. For near-bed water samples, a 2.2-L stainless steel Kemmerer water sampler was deployed at each location to obtain water samples six inches above the sediment bed. A 150-mL sample was then decanted into a pre-cleaned, acid-washed 200-mL polyethylene bottle. Samples were then placed on ice during transportation and stored at −20℃ until analysis. Sediment samples (1–4 cm deep) were obtained using a van veen grab sampler from 21 sites, transported on ice, and desiccated for 48 h in a 100℃ gravity-drying oven. Dried samples were then disaggregated using a mortar and pestle and sieved to 63 μm. One gram of processed sample was digested in 20 mL aqua regia (trace metal grade 1HNO_3_/3HCl/3H_2_O) at 40 °C for 4 h, diluted to 100 mL with nanopure ultra-deionized water, and stored in acid-washed 125 mL Nalgene® bottles. Aqua regia digestion is widely recognized as a partial digestion method that extracts the environmentally available and acid-soluble fraction of metals rather than total concentrations (Rauret et al., 1999). This approach is appropriate for environmental risk assessment indices such as Igeo and PERI, which are designed to evaluate anthropogenic enrichment and bioavailable fractions rather than total lithogenic metal content (Förstner & Wittmann, 2012; Sutherland, 2000). The concentrations of aluminum (Al), As, boron (B), Cd, Co, Cr, Cu, iron (Fe), magnesium (Mg), Mn, Ni, Pb, and Zn were analyzed in triplicate using argon ICP-OES (inductively coupled plasma optical emission spectroscopy, Thermoscientific iCAP 6300) at wavelengths chosen to reduce potential spectral interferences. Standards for each element were purchased from Spex Certiprep (Metuchen, NJ) and diluted to appropriate concentrations using nanopure ultra-deionized water. Standard curves for each element yielded correlation coefficients ranging from 0.998 to 1.000. Quality assurance and quality control procedures included the analysis of procedural blanks (Milli-Q ultrapure water) and field blanks. Procedural blanks were included with each digestion batch to assess potential contamination during sample preparation. Method detection limits (MDLs) were determined based on standard deviations of replicate blanks. Analytical precision, assessed through triplicate measurements, yielded relative standard deviations < 10%.

### Statistical analyses

All analyses were conducted using R version 4.5.2. All raw data for metal concentrations found in sediment, surface water, and near-bed water samples have been provided in supplement A.

### Heavy Metal Pollution Index (HPI)

To calculate the HPI, drinking-water guideline values were compiled for Al, As, Cd, Cr, Cu, Fe, Mn, Ni, and Pb using the USEPA Primary and Secondary Maximum Contaminant Levels and World Health Organization drinking-water guideline values. Unit weights (W_*i*_) were calculated using the formula:

$$W_{\mathcal i\;}=\frac1{S\mathcal i}$$

where *S*_*i*_ is the standard permissible limit provided by various regulatory sources (Table 2).

**Table 2:** Maximum contaminant level or treatment technique standards used for HPI calculations

Analyte	Maximum contaminant level or treatment technique standard (µg/L)	Source
Al	200	USEPA Secondary Drinking Water Standards^1^
As	10	USEPA National Primary Drinking Standards^2^
Cd	5	USEPA National Primary Drinking Standards^2^
Cr	100	USEPA National Primary Drinking Standards^2^
Cu	1300	USEPA National Primary Drinking Standards^2^
Fe	300	USEPA Secondary Drinking Water Standards^1^
Mn	50	USEPA Secondary Drinking Water Standards^1^
Ni	70	WHO^3^
Pb	10	USEPA National Primary Drinking Standards^2^

^1^(United States Environmental Protection Agency, 2025)

^2^(U.S. Environmental Protection Agency, 2025), ^3^(World Health Organization, 2017)

In this study, *S*_*i*_ values were derived from United States Environmental Protection Agency drinking water standards and, where applicable, World Health Organization guideline values. Although these standards are not specifically designed for ecological risk assessment, their application in HPI calculations for surface water and field-collected samples is well established in the literature as a conservative screening approach. Additionally, the use of drinking water standards has been previously applied to various large-scale studies examining trace metal contamination in surface waters (Kumar et al., 2019; Le et al., 2025; Tiwari et al., 2015).

Sub-index quality ratings (*Q*_*i*_) were then calculated using the formula:

$$Q_i\;=\;\;100\times\left(\;\left(M_i-I_i\right)\;/\;\left(S_{i\;}-\;I_i\right)\right)\;=\;100\;\times\;Mi/S_{i\;}$$

where *M*_*i*_ is the measured concentration and *I*_*i*_ is the ideal value (0). For this equation, *Q*_*i*_ = 100 when *M*_*i*_ = *S*_*i*_, *Q*_*i*_ > 100 when the standard was exceeded. HPI for each site was calculated using the following equation:

$$HPI\;=\;{\textstyle\sum_{i=1}^n}\;W_iQ_{i\;}/\;{\textstyle\sum_{i=1}^n}{\textstyle\;}{\textstyle W_i}$$

Sites were then categorized based on the interpretation system outlined in Table 3.

**Table 3:** Categories used for interpretation of HPI

HPI range	Category
0–25	Very low: most metals far below limits
25–50	Low-moderate: some metals approaching limits
50–100	Elevated: at least one metal near/over standards or several moderately high
> 100	Critical pollution: unacceptable overall exceedance pressure above guidelines

### Geoaccumulation Index (Igeo)

*I*geo calculations were performed using the following equation:

$$Igeo\;=\;\log_2\left(C/1.5B_n\right)$$

where C denotes the actual measured concentration for each metal, B_n_ represents the background concentration, and the constant of 1.5 represents natural fluctuations in the content of a given substance in the environment with minimal human influence. Background concentrations (Al—19,700 ppm, As—1.8 ppm, Cd—0.1 ppm, Cu—4.1 ppm, Fe—6000 ppm, Li—8 ppm, Mg—900 ppm, Mn—234 ppm, Ni—3.4 ppm, Pb—9.9 ppm, and Zn—22 ppm) were obtained from reference data provided by the United States Geological Service (Smith et al., 2013) (site C-350597 (Code 5820)). The use of regional background values is preferred over global averages as it accounts for local lithological variability and improves the accuracy of anthropogenic enrichment assessments (Reimann & de Caritat, 2005). Reference site selection was based on geographical location compared to sampling locations, level of urbanization, and primary/secondary landcover types. *I*_*geo*_ values were then converted into standard Müller classes (Table 4) to allow for the interpretation of results.

**Table 4:** Müller classes and interpretations used for *I*_*geo*_ index values

*I*_*geo*_ range	Class	Interpretation
≤ 0	0	Uncontaminated
0–1	1	Uncontaminated to moderately contaminated
1–2	2	Moderately contaminated
2–3	3	Moderately to heavily contaminated
3–4	4	Heavily contaminated
4–5	5	Heavily to extremely contaminated
> 5	6	Extremely contaminated

### Potential Ecological Risk Index (PERI)

PERI calculations were performed using Cd, Cu, Pb, Ni, Cr, and Zn. Using the same background values described in the *I*_*geo*_ methods above, contamination factors (*C*_*f*_) were first calculated using the following equation:

$$C_f\;=\;C_i/C_o$$

where *C*_*i*_ is the measured concentration of a given metal and *C*_*o*_ is the background concentration. Potential ecological risk factors (Er) were then calculated using the following equation:

$$E_r=T_{r\;}\times\;C_{f\;}$$

where *T*_*r*_ for Cd = 30; Cu = Pb = Ni = 5; Cr = 2; Zn = 1. *T*_*r*_ values for Cd, Cu, Pb, Cr, and Zn were based on recommendations provided in Hakanson (1980). Ni was based on modifications described in Li et al. (2024).

Finally, the overall PERI for each site was calculated using

$$PERI=\Sigma\mathrm{E}_r^i$$

Overall PERI values were then assigned a class based on the categories listed below (Table 5).

**Table 5:** PERI cumulative risk classifications as described in Hakanson (1980)

Overall PERI cumulative value	PERI class
< 150	Low
150–300	Moderate
300–600	Considerable
≥ 600	High

### Multivariate cluster analysis

To identify potential associations of trace metal concentrations in surface water, near-bed water, and sediment samples with munitions testing, multivariate cluster analyses were conducted. Due to the high amount of variation between metals, clustering computations were performed in standardized space using *z*-score normalization across sites to ensure equal weighting (Everitt & Hothorn, 2011). First, standardized values (*z*_*ij*_) were generated using the formula:

$$Z_{ij\;}=\;\frac{x_{ij}\;-\mu_j}{\sigma_j}$$

where ${x}_{ij}$ is the concentration at site $i$, ${\mu }_{j}$ is the mean standard deviation of analyte *j*, and ${\sigma }_{j}$ is the population standard deviation of analyte $j$ across all sites. Variables with zero variance across sites were assigned *z*-scores of 0 to avoid division by zero. Inter-site dissimilarity was quantified using Euclidean distance in standardized space using

$$d\left(i,k\right)\;=\;\vert\vert\;z_i\;-\;z_{k\;}\vert\vert_2$$

where *z*_*i*_ and *z*_*k*_ are the standardized eleven-element vectors for sites *i* and *k*. To identify the optimal number of clusters for each sample medium, the silhouette method was utilized (Rousseeuw, 1987) to evaluate candidate values from two to eight with the upper bound constrained by *n* −1 (where *n* is the number of sites for that medium) using the equation below:

$$s\left(i\right)\;=\;\frac{b\left(i\right)\;-\;a\left(i\right)}{max\;\left\{a\left(i\right),\;b\left(i\right)\right\}}$$

where *a*(*i*) is the mean distance from site *i* to all other sites in an assigned cluster and *b*(*i*) is the minimum distance from site *i* to sites in any other cluster. The optimal number of clusters was based on the value of *k* that maximized the mean silhouette coefficient across all sites (Rousseeuw, 1987). Final cluster assignments were produced using Ward’s minimum-variance linkage (Ward Jr., 1963) and visualized spatially by linking each sampling site’s cluster assignment to its geographic coordinates (latitude/longitude) using R packages sf, ggplot2, ggspatial, dplyr, tibble, and patchwork.

## Results

### Surface and near-bed water samples

**Fig. 3:**
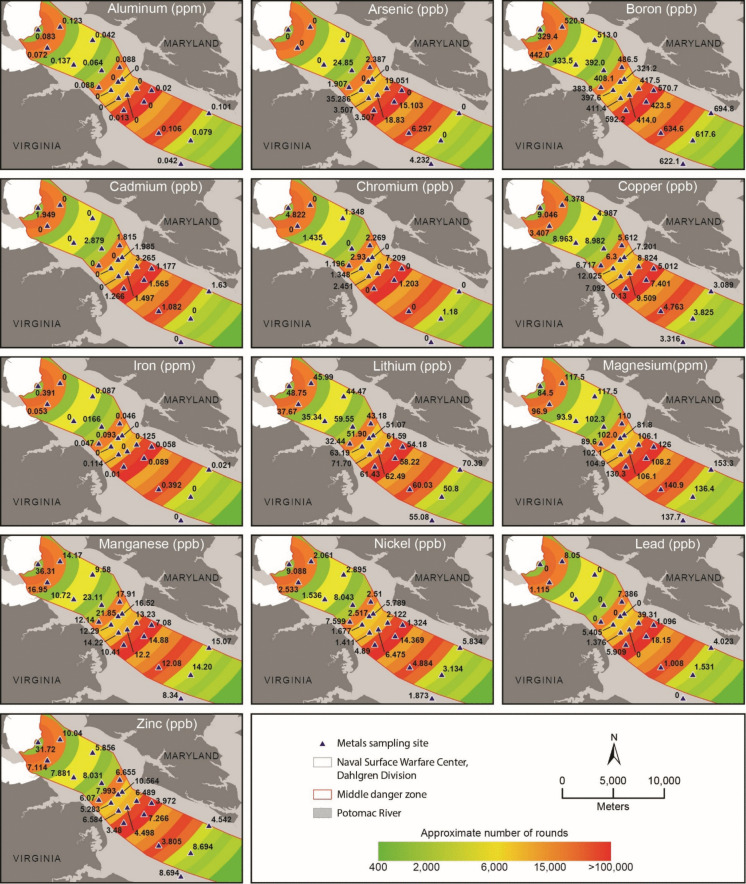
Metal concentrations for surface water samples quantified using ICP-OES

**Fig. 4:**
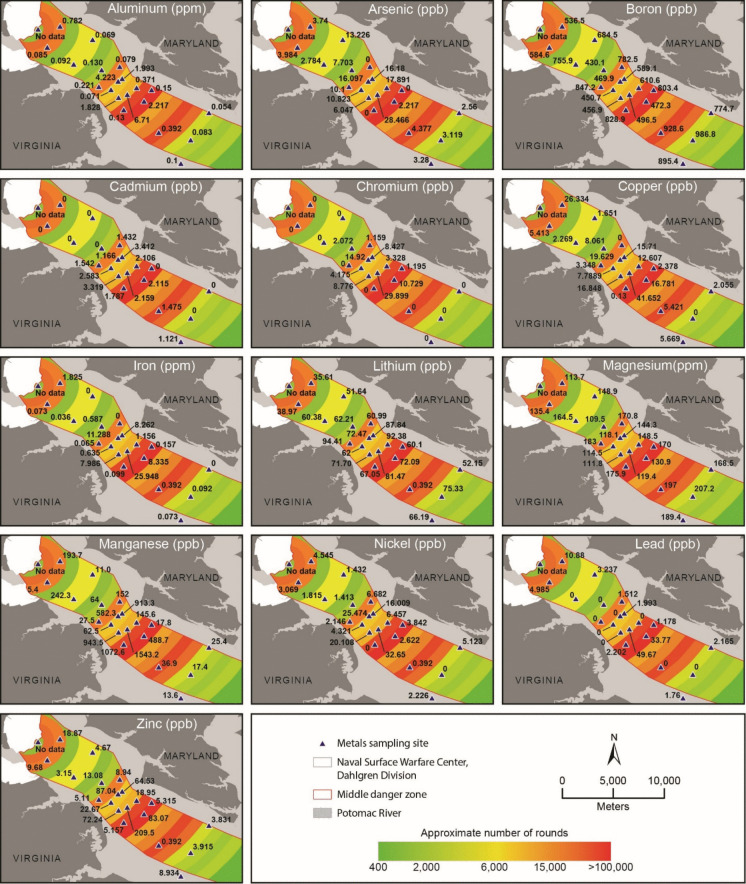
Metal concentrations for near-bed water samples quantified using ICP-OES

The measured concentrations of all trace metals sampled are provided in Figs. 3 and 4. For surface waters, the highest observed concentrations of Fe (391 ppb), Mn (36.305 ppb), and Zn (31.719 ppb) were found in site 1 which is located immediately offshore of the Naval Surface Warfare Center Dahlgren Division. Arsenic (35.286 ppb) and Cu (12.025 ppb) were found at the highest concentrations in site 11, and site 12 had the highest concentrations of Cd (3.265 ppb), Cr (7.209 ppb), and Pb (39.309 ppb). Maximum concentrations of B (694.8 ppb), Li (70.39 ppb), and Mg (153.3 ppm) were observed in site 19.

Near-bed water samples had the highest concentrations of Cr (28.899 ppb), Cu (41.652 ppb), Fe (29.498 ppb), Mn (1543.42 ppb), Ni (32.65 ppb), Pb (49.628 ppb), and Zn (209.51 ppb) in site 13. Boron (986.8 ppb) and Mg (207.23 ppm) were highest in site 20, Cd at site 9 (3.412 ppb), and Li at site 8 (94.41 ppb).

### HPI

Of the 21 surface water sites tested, twelve returned HPI indices classified as “very low” and ranged from 1.4507 to 22.3305 (Table 6). Sites 7 (HPI = 39.9101), 17 (HPI = 25.4377), 18 (HPI = 28.0555), and 19 (HPI = 25.4547) were classified as “moderate pollution”. Sites 6 (HPI = 83.369), 11 (HPI = 90.9394), 13 (56.1077), and 16 (89.3509) were elevated with at least one metal near or over standards. One site (site 12 = 159.2542) was classified as “critically polluted”. Arsenic concentrations were found to exceed standards in sites 6, 11, 12, 13, and 16, and Pb standards were exceeded in sites 12 and 16. For sites classified as elevated or critical, As (sites 6, 11, 13) and Pb (sites 12 and 16) were identified as the top driver for reported HPI indices.

**Table 6:** Surface water HPI results

Site	HPI	Category	Metals exceeding standards	Top driver
1	22.3305	Very low	Fe	Cd
2	19.7769	Very low	None	Pb
3	4.6852	Very low	None	Pb
4	1.4507	Very low	None	Mn
5	1.8032	Very low	None	Mn
6	83.369	Elevated	As	As
7	39.9101	Low-moderate	None	Pb
8	6.3135	Very low	None	As
9	19.2317	Very low	None	Cd
10	2.3345	Very low	None	Mn
11	90.9394	Elevated	As	As
12	159.2542	Critical pollution	As, Pb	Pb
13	56.1077	Elevated	As	As
14	12.4101	Very low	None	As
15	13.7387	Very low	None	Cd
16	89.3509	Elevated	As, Pb	Pb
17	25.4377	Low-moderate	None	Pb
18	28.0555	Low-moderate	None	As
19	25.4547	Low-moderate	None	Cd
20	5.2328	Very low	None	Pb
21	10.3863	Very low	None	As

Compared to surface waters, near-bed water samples exhibited a substantially higher degree of contamination. Six sites (3, 6, 15, 19, 20, and 21) were classified as “very low,” with HPI values ranging from 5.5774 to 24.902 (Table 7). Sites 4 (37.7271), 5 (28.1921), 7 (30.1289), 8 (39.7811), and 18 (29.5320) were classified as “low–moderate,” indicating increasing influence of metal inputs relative to background conditions. Sites 2 (58.329), 11 (54.410), and 12 (76.135) were categorized as “elevated,” reflecting the presence of one or more metals approaching or exceeding guideline values. Six sites (9, 10, 13, 14, 16, and 17) were classified as “critically polluted,” with HPI values ranging from 116.2143 to 430.1786. Exceedances were most frequently associated with Al, Fe, Mn, As, and Pb, consistent with mobilization of redox-sensitive metals in near-bed environments (Charlatchka & Cambier, 2000; Xie et al., 2019). Among elevated and critical sites, Mn (5/9 sites), Pb (2/9 sites), and As (2/9 sites) were identified as the dominant contributors to HPI values (Table 7).

**Table 7:** Near-bed water HPI results

Site	HPI	Category	Metals exceeding standards	Top driver
1	Sample lost during transport			
2	58.32905	Elevated	Al, Fe, Mn, Pb	Pb
3	21.04678	Very low	None	Pb
4	37.72715	Low-moderate	As	As
5	28.19213	Low-moderate	Mn	Mn
6	24.90201	Very low	Fe, Mn	As
7	30.139	Low-moderate	Mn	Mn
8	39.78118	Low-moderate	Al, As	As
9	182.7714	Critical pollution	Al, As, Fe, Mn	Mn
10	149.5876	Critical pollution	Al, As, Fe, Mn	Mn
11	54.41023	Elevated	As, Fe, Mn	As
12	76.13475	Elevated	Al, As, Fe, Mn	As
13	430.1786	Critical pollution	Al, As, Fe, Mn, Pb	Mn
14	156.6069	Critical pollution	Al, Fe, Mn	Mn
15	5.577403	Very low	None	Pb
16	185.042	Critical pollution	Al, Fe, Mn, Pb	Pb
17	116.2143	Critical pollution	Mn	Mn
18	29.53202	Low-moderate	Al, Fe	Cd
19	13.19019	Very low	None	As
20	9.095768	Very low	None	As
21	23.03737	Very low	None	Cd

### Sediments

**Fig. 5:**
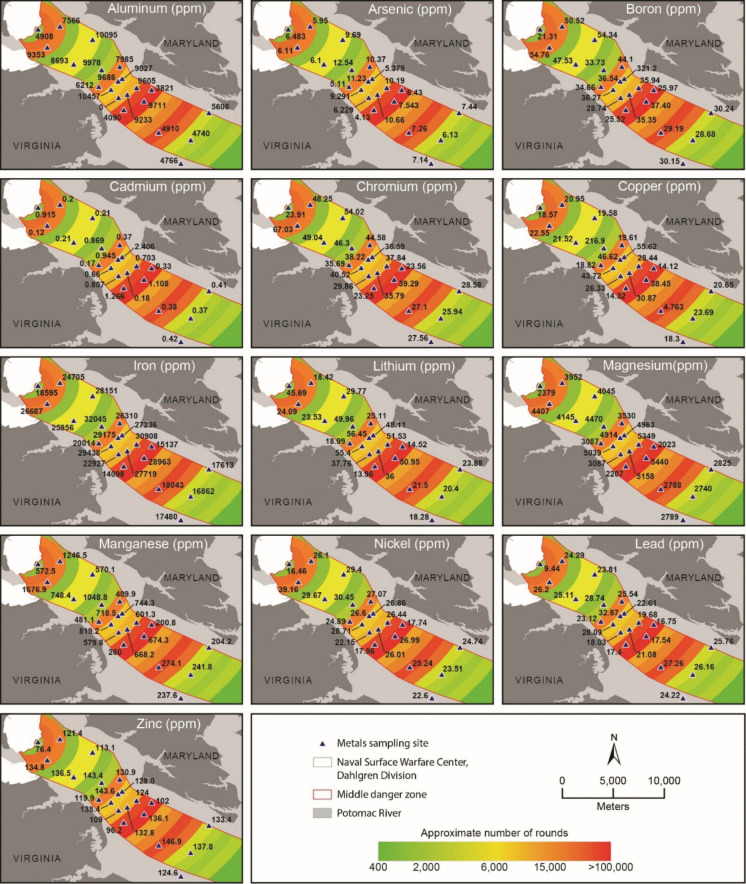
Metal concentrations for sediment samples quantified using ICP-OES

Measured sediment concentrations of all metals are provided in Fig. 5. The highest concentration of B (54.76 ppm), Cr (67.03 ppm), Mn (1676.9 ppm), and Ni (29.16 ppm) was observed in site 3. Site 6 contained the highest concentrations of As (12.538 ppm), Cu (216.98 ppm), Fe (32,045 ppm), and Pb (28.74 ppm). Maximum concentrations of Cd (2.406 ppm), Li (56.45 ppm), Al (10,457 ppm), Mg (5440 ppm), and Zn (146.87 ppm) were detected in sites 9, 10, 11, 16, and 18, respectively.

### I_geo_

Contamination at the sampled sites appears consistent with anthropogenic influences described in previous studies. Across the 231 site–metal classifications (21 sites × 11 metals), only 3.46% of observations were Class 0 (unpolluted), with most values concentrated in Classes 1–3 (26.84% Class 1, 45.89% Class 2, and 22.51% Class 3) (Table 8). Categories 4, 5, and 6 were rare occurrences (0.43% each) and were restricted to Cd and Cu. Arsenic enrichment was limited, reaching Class 3 only at sites 6 and 10. Cadmium was present at categories greater or equal to 3 at sites 1, 6, 9, 10, 11, 12, 13, 14, and 16, while Cu was elevated at sites 6, 9, 10, 11, 12, 13, 14, and 16. Lithium also showed elevated classes at sites 6, 9, 10, 11, 12, and 16. Nickel was ubiquitously elevated across all sites sampled (class 2 or 3).

**Table 8:** I_geo_ results

Site	As	Cd	Cr	Cu	Fe	Li	Mg	Mn	Ni	Pb	Zn
1	2	3	1	2	2	2	1	1	2	0	2
2	2	1	2	2	2	1	2	2	3	1	2
3	2	0	2	2	2	2	2	3	3	1	3
4	2	1	2	2	2	2	2	1	3	1	2
5	2	1	2	2	2	1	2	2	3	1	3
6	3	3	2	6	2	3	2	2	3	1	3
7	2	2	2	2	2	2	2	1	3	1	2
8	1	1	1	2	2	1	2	1	3	1	2
9	1	5	2	4	2	3	2	2	3	1	2
10	3	3	2	3	2	3	2	2	3	2	3
11	2	3	2	3	2	3	2	2	3	1	3
12	2	3	2	3	2	3	2	1	3	1	2
13	2	3	1	3	2	2	2	1	3	1	3
14	2	3	1	3	2	2	2	1	3	1	2
15	2	2	1	2	1	1	1	0	2	1	2
16	2	3	2	3	2	3	3	1	3	1	3
17	1	1	1	2	1	1	1	0	2	1	2
18	2	2	1	2	2	1	2	0	3	1	3
19	2	2	1	2	1	1	2	0	3	1	3
20	2	2	1	2	1	1	2	0	3	1	3
21	2	2	1	2	1	1	2	0	3	1	2

### PERI

PERI results are presented in Table 9. Only three locations sampled (sites 2, 8, and 17) were categorized as low risk. Sites 3, 4, 5, 7, 15, 18, 19, 20, and 21 were classified as moderate risk with PERI values ranging from 151.6 to 208.4. Locations identified as having considerable risk included sites 1, 6, 10, 11, 12, 13, 14, and 16. Only one location (site 9) was identified as having a high risk category with a relatively high PERI value of 852.5. With the exceptions of sites 1 and 6, all sites categorized as either considerable or high risk (9, 10, 11, 12, 13, 14, and 16) are in areas identified as having the heaviest distribution of large-caliber projectiles. Due to its relatively high toxicity and concentration compared to the other included metals, Cd was the largest driver of PERI values for all sites except for site 3 (Ni) and 6 (Cu).

**Table 9:** PERI results

Site	Cd	Cr	Cu	Ni	Pb	Zn	PERI	PERI category	Top driver
1	274.5	4.0	22.6	24.2	4.8	3.5	333.6	Considerable	Cd
2	60	8.0	25.5	38.4	12.3	5.5	149.8	Low	Cd
3	36	11.2	27.5	57.6	13.2	6.1	151.6	Moderate	Ni
4	63	9.0	23.9	43.2	12.0	5.1	156.3	Moderate	Cd
5	63	8.2	26.2	43.6	12.7	6.2	159.9	Moderate	Cd
6	260.7	7.7	264.6	44.8	14.5	6.5	598.8	Considerable	Cu
7	111	7.4	23.9	39.8	12.9	6.0	201.0	Moderate	Cd
8	51	5.9	23.0	36.6	11.7	5.5	133.6	Low	Cd
9	721.8	6.1	67.8	39.5	11.4	5.8	852.5	High	Cd
10	283.5	6.4	56.9	39.1	16.6	6.5	409.0	Considerable	Cd
11	198	6.8	53.3	42.2	14.2	6.3	320.8	Considerable	Cd
12	210.9	6.3	34.7	38.9	9.9	5.6	306.3	Considerable	Cd
13	261.9	6.0	37.6	38.3	10.6	6.0	360.4	Considerable	Cd
14	242.1	5.0	32.1	32.6	9.1	5.0	325.8	Considerable	Cd
15	99	3.9	17.2	26.1	8.5	4.6	159.3	Moderate	Cd
16	332.4	6.5	46.9	39.7	8.9	6.2	440.6	Considerable	Cd
17	54	3.9	17.5	26.4	8.8	4.4	115.0	Low	Cd
18	114	4.5	26.1	37.1	13.8	6.7	202.2	Moderate	Cd
19	123	4.8	25.2	36.4	13.0	6.1	208.4	Moderate	Cd
20	111	4.3	28.9	34.6	13.2	6.3	198.3	Moderate	Cd
21	126	4.6	22.3	33.2	12.2	5.7	204.0	Moderate	Cd

### Multivariate cluster analyses

**Fig. 6:**
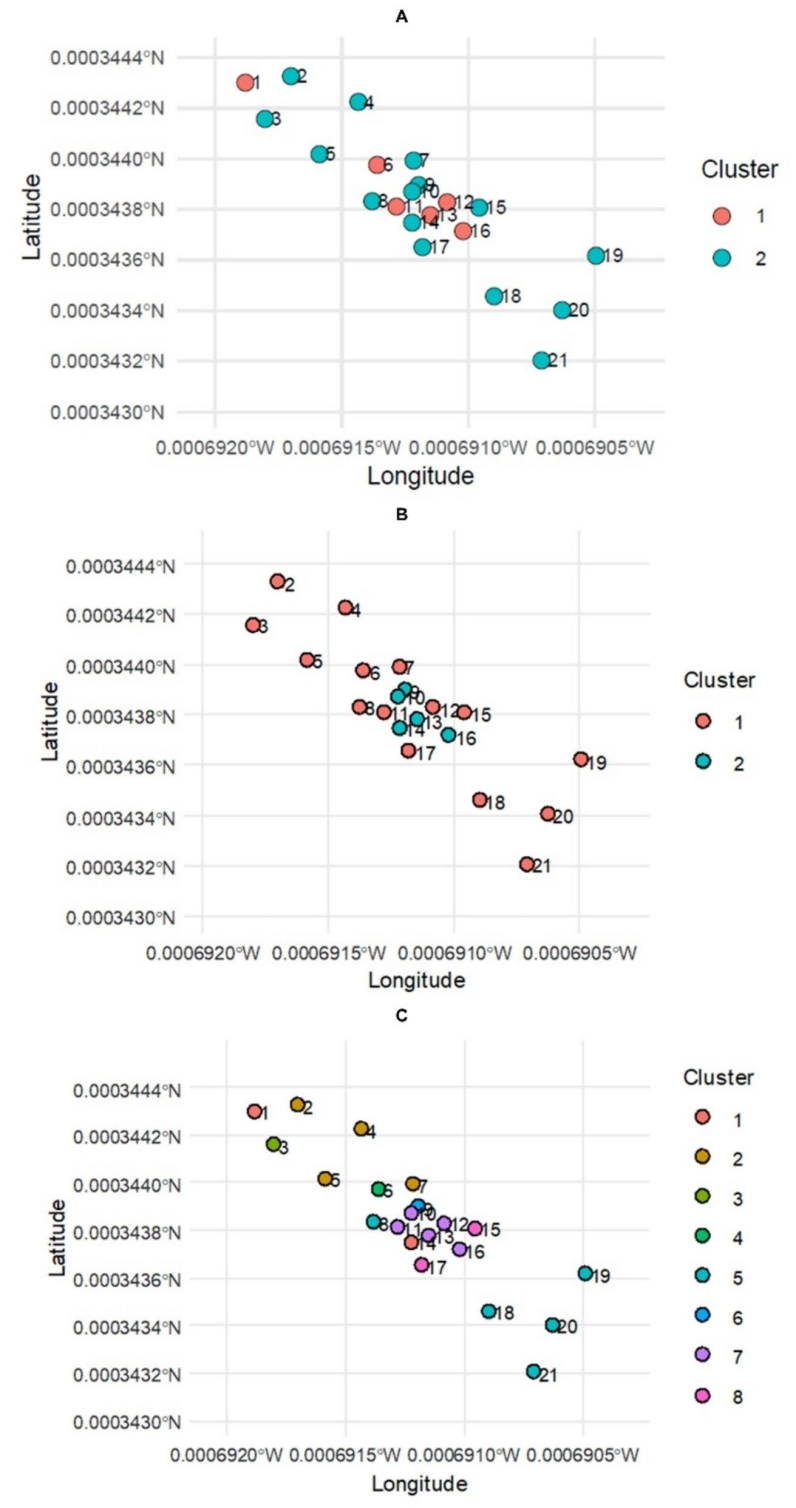
Multivariate cluster assignments produced using Ward’s minimum-variance linkage for **A** surface water, **B** near-bed water, and **C** sediments

Based on the results of the silhouette method, the optimal number of clusters was *k* = 2 for surface water samples, *k* = 2 for near-bed water samples, and *k* = 8 for sediment samples. For both surface and near-bed water samples, site clustering was spatially aligned with the shoreline of the Dahlgren Naval Facility and the heaviest distribution of large-caliber projectiles in the middle danger zone (*n* = 5) compared to low density zones (*n* = 15) (Fig. 6A). Similar results were observed for near-bed water, with sites 9, 10, 13, 14, and 16 clustering together (Fig. 6B). Sediments were more complex, with sites 3, 6, 8, 9, and 16 identified as singleton clusters (Fig. 6C). The remaining sites were roughly distributed based on location above (2, 4, 6, and 7), inside (10, 11, 12, 13, and 16; 15 and 17), and below (18, 19, 20, and 21) the heaviest distribution of large-caliber projectiles.

## Discussion

Study limitations included the inability to collect samples during or immediately following live testing events which may have eliminated detection of short-lived concentration spikes in surface and near-bed waters and the inability to assess the presence and concentrations of organic constituents associated with munitions testing which could serve as important specific environmental markers. Additionally, temporal variability associated with hydrodynamic conditions (tidal cycles, re-suspension, etc.) was not explicitly included in the study design and may influence observed metal distributions in matrices.

Surface water contamination was found to be limited, with 12 locations identified as “very low” based on HPI values and one site identified as “critical”. In contrast, near-bed water samples exhibited greater contamination, with nine sites classified as either “elevated” (*n* = 3) or “critical” (*n* = 6), indicating stronger influence of sediment–water interactions on dissolved metal concentrations. Elevated metal concentrations in near-bed waters are consistent with well-established sediment–water exchange processes and lower concentrations found in surface waters may be due to dilution effects from river flow. Metals can be released from sediments under changing redox conditions, particularly when reducing environments promote the dissolution of Fe- and Mn-oxyhydroxides that act as important sorbents for trace metals (Charlatchka & Cambier, 2000; Hatje et al., 2003). Fluctuating redox conditions driven by organic matter degradation and sediment oxygen demand can also enhance the mobilization of metals into the overlying water column in estuarine systems (Burton, 2018; Eggleton & Thomas, 2004). Physical disturbance of sediments from physical disturbances (Kalnejais et al., 2010) and bioturbation (Xie et al., 2019) can further influence the transport and fate of trace elements across the sediment–water interface.

Based on I_geo_ and PERI values, sediment metal contamination from anthropogenic sources remains widespread within the Potomac River, with only three sites (2, 8, and 17) classified as having low ecological risk. The persistence of elevated sediment concentrations reflects the role of fine-grained sediments as long-term sinks for trace metals, particularly in slower-flowing depositional environments where metals are associated with organic matter, sulfides, and Fe–Mn oxide phases (Förstner & Wittmann, 2012). However, these sediments can also serve as secondary sources of contamination when environmental conditions shift, reinforcing the importance of considering both storage and remobilization processes in risk assessments. We observed that concentrations of metals have appeared to decrease when comparing sites 19 and 20 with previous data collected in 2002 from two geographically close locations (Table 10).

**Table 10:** Comparison of trace sediment contamination found in this study vs. data from the 2013 Water Range Sustainability Environmental Program Assessment (Naval Surface Warfare Center, 2013)

	**Sediment metal concentration (ppm)**						
**Site**	**Cd**	**Cr**	**Cu**	**Pb**	**Mn**	**Ni**	**Zn**
864 (MDZ)^1^	0.703	90.5	46.6	40.5	648	51.8	206
Site 19	0.41	28.58	20.65	25.76	204.2	24.74	133.42
867 (MDZ)^1^	0.764	88.1	49.1	42.8	735	52.3	215
Site 20	0.37	25.94	23.69	26.16	241.8	23.51	137.76

^1^Naval Surface Warfare Center (2013)

Although slight differences in extraction efficiency may occur between aqua regia digestion and the USEPA strong-acid digestion methods (e.g., 3050B) used in the Water Range Sustainability Environmental Program Assessment, both approaches quantify operationally defined “total recoverable” metal concentrations and are considered comparable for environmental assessment and index-based analyses. The observed declines potentially suggest a combination of increased regulatory scrutiny, shifts in industrial activity away from the watershed, and improved management and remediation practices that reduce sediment loads (Noe et al., 2020; Owens & Cornwell, 1995; Schueler & Youngk, 2015). In addition, long-term sediment burial, dilution through sediment accretion, and downstream transport processes may contribute to observed decreases in surface sediment concentrations (Walling, 2006).

**Fig. 7:**
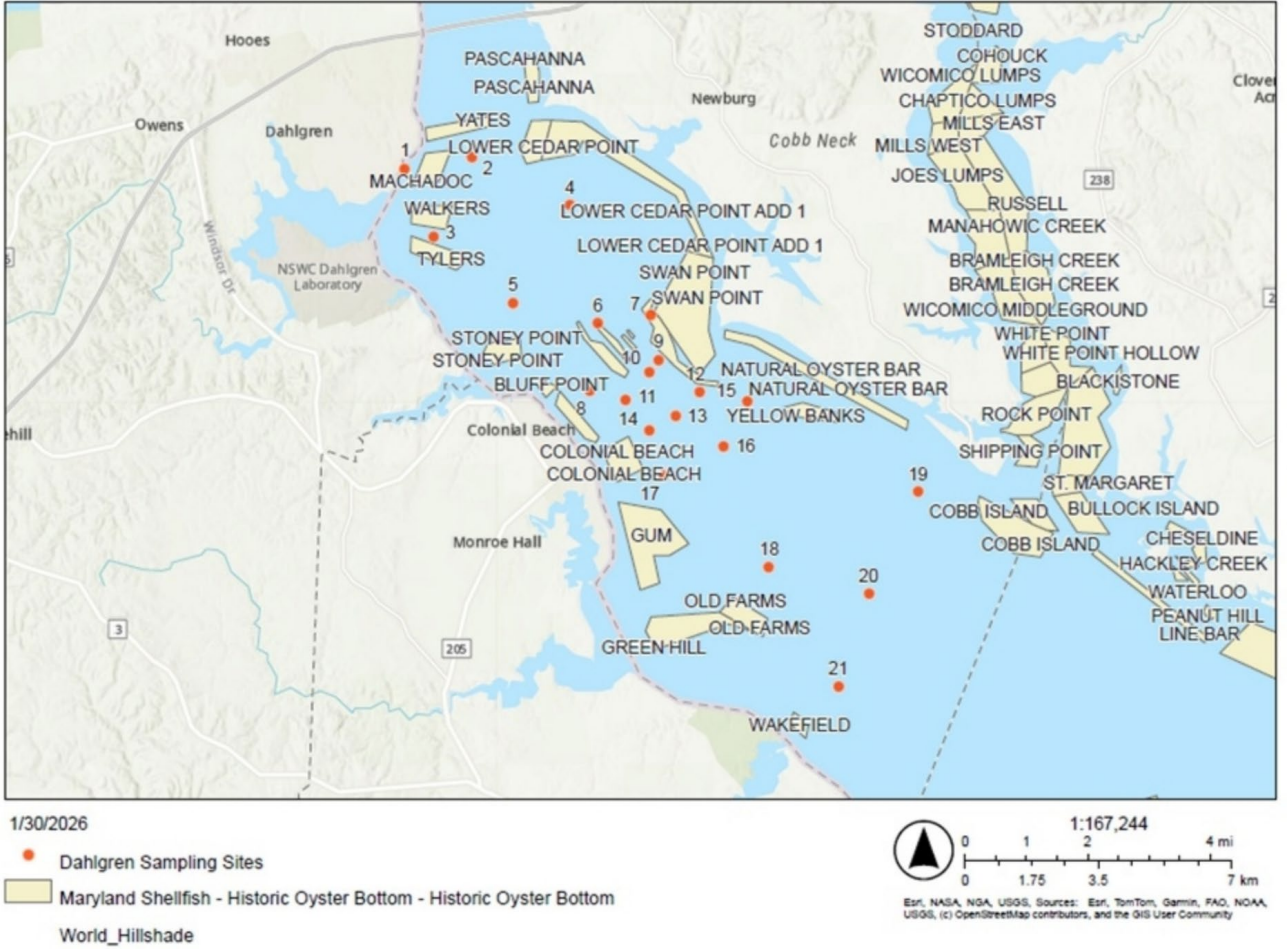
Sampling locations relative to historic oyster beds. Modified from Maryland Department of Natural Resources (2016)

Multiple historic oyster (*Crassostrea virginica*) bars occur within or adjacent to the sampled areas (Fig. 7), including sites where elevated HPI, Igeo, and PERI values were observed.

Given that near-bed waters and sediments represent primary exposure pathways for benthic and filter-feeding organisms, the elevated concentrations of Mn, Pb, Cd, and Cu identified in these zones are directly relevant to ecological risk. *C. virginica* has been shown to bioaccumulate trace metals from both water and sediments (Apeti et al., 2005; Rodney et al., 2007) and has been widely utilized as a biomonitoring species (Aguilar et al., 2012). Concentrations of Cd, Cr, and Cu at or above those found in several sites have been shown to cause lysosomal destabilization (Aguilar et al., 2012), suppressed hemocyte metabolism and immune-related functions (Ivanina et al., 2014), and disrupted embryonic development (Calabrese et al., 1973; MacInnes & Calabrese, 1979) in *C. virginica*. Similarly, this reach of the Potomac River provides critical habitat for Atlantic sturgeon (*Acipenser oxyrinchus*), a benthic-associated species vulnerable to sediment-associated contaminants (National Oceanic and Atmospheric Administration 2022). Elevated metal concentrations in near-bed waters and sediments suggest that biologically relevant exposure pathways coincide spatially with contamination hotspots identified in this study. This linkage strengthens the ecological relevance of the HPI, Igeo, and PERI results beyond general background context.

As noted in previous studies, the Potomac River has been impacted by a complex mixture of point and non-point contamination sources (Sapp et al., 2024), complicating source attribution. Hydrodynamic processes characteristic of estuarine systems play a critical role in controlling contaminant transport, deposition, and resuspension (Uncles et al., 2002). These processes can create spatial heterogeneity in sediment accumulation and contaminant distribution, particularly in transitional zones between freshwater and estuarine conditions. However, several lines of evidence suggest a strong association between munitions testing and observed contamination patterns. Of the top 30 inorganic constituents identified by the 2013 Water Range Sustainability Environmental Program Assessment, Cu, Mn, Zn, Pb, Cr, and Cd were selected for fate and transport modeling due to their total mass of constituents contained in projectiles and relative toxicity of each constituent (Naval Surface Warfare Center 2013). Sites with elevated HPI values in both surface and near-bed waters were consistently located within the primary target area or dense projectile zones where hydrodynamic conditions may promote localized deposition of particulate-bound metals. Additionally, areas with high projectile density may contribute to the localized enrichment of metals such as Pb, Cu, and Mn through corrosion and fragmentation. The co-occurrence of elevated concentrations across multiple indices (HPI, Igeo, and PERI) within these zones strengthens the inference of a munitions-related signal, despite the presence of broader watershed-scale contamination. In surface waters, Pb was the primary driver of “elevated” HPI classifications in 2 of 5 elevated/critical sites (40%) and was the sole driver at the single “critical” site. In near-bed waters, 9 sites were categorized as either elevated or critical; of these, Mn was the dominant contributor in 5 sites (55.6%), Pb in 2 sites (22.2%), and As in 2 sites (22.2%). These patterns are consistent with known geochemical behavior of these elements where Pb is strongly particle-associated, Mn is highly redox-sensitive, and Cd exhibits high ecological risk due to its toxicity coefficient in PERI frameworks (Eggleton & Thomas, 2004). Multivariate cluster analyses produced using Ward’s minimum-variance linkage revealed a clear distinction for sites located in the dense zone for surface and near-bed water samples, and analysis of sediment data revealed relationships between sites located above, inside, and below the dense zone.

## Conclusions

This work provides one of the first field-based, multi-matrix assessments of trace metal contamination in the PRTR and directly links observed contamination patterns with projectile-density distributions. By integrating water and sediment analyses with established ecological risk indices, this study advances current understanding of contaminant behavior in active military testing environments and offers a transferable framework for evaluating contamination in other data-limited aquatic systems influenced by legacy munitions. The identification of contamination hotspots within primary target and dense-use zones highlights the need for targeted monitoring and potential mitigation strategies in areas of elevated ecological risk. These findings are critical for protecting sensitive habitats within the Potomac River watershed and developing public health guidance related to recreational and subsistence use of the river. Future research should focus on (1) high-frequency or event-based sampling to capture transient contamination pulses, (2) inclusion of organic munitions-related compounds to improve source specificity, (3) assessment of metal bioavailability and uptake in aquatic organisms, and (4) development of coupled hydrodynamic-geochemical models to better predict contaminant transport and fate under changing environmental conditions.

## Supplementary Information

Below is the link to the electronic supplementary material.

ESM 1 (PDF 63.9 KB)

## Data Availability

Data from this study has been provided as supplemental material A.
