# Supplementary material for: Assessing the presence, concentrations, and potential ecological impacts of trace metal contamination in the Potomac River Test Range Complex middle danger zone
Source: Environ Monit Assess. 2026 May 1;198(5):535. doi: 10.1007/s10661-026-15382-2 (PMC13134989; doi:10.1007/s10661-026-15382-2)
Supplement: Supplementary file 1 — (PDF 63.9 KB) [file 10661_2026_15382_MOESM1_ESM.pdf]

Surface Water Trace Metal Data (ICP-OES)

Site	Latitude	Longitude	Al [ppm]	As [ppb]	B [ppb]	Cd [ppb]	Cr [ppb]	Cu [ppb]	Fe [ppm]	Li [ppb]	Mg [ppm]	Mn [ppb]	Ni [ppb]	Pb [ppb]	Zn [ppb]
1	38.32762	-77.0197	0.08	0.00	329.36	1.95	4.82	9.05	0.39	48.75	84.46	36.31	9.09	0.00	31.72
2	38.33036	-76.9997	0.12	0.00	520.94	0.00	0.00	4.38	0.00	45.99	117.54	14.17	2.06	8.05	10.04
3	38.31157	-77.0111	0.07	0.00	441.98	0.00	0.00	3.41	0.05	37.67	96.92	16.95	2.55	1.16	7.11
4	38.31897	-76.9702	0.04	0.00	513.02	0.00	1.35	4.99	0.09	44.47	117.48	9.58	2.90	0.00	5.86
5	38.29606	-76.9872	0.14	0.00	433.52	0.00	1.44	8.96	0.00	35.34	93.91	10.72	1.54	0.00	7.88
6	38.29129	-76.9618	0.06	24.85	392.01	2.88	0.00	8.98	0.17	59.55	102.33	23.11	8.04	0.00	8.03
7	38.2932	-76.9458	0.09	2.39	486.47	1.82	2.27	5.61	0.05	43.18	109.97	17.91	2.51	7.39	6.66
8	38.27543	-76.964	0.10	1.91	383.82	0.00	1.97	6.72	0.05	32.44	89.64	12.14	7.60	0.00	6.07
9	38.2826	-76.9436	0.00	0.00	321.24	1.99	0.00	7.20	0.00	51.07	81.84	16.52	5.79	0.00	10.56
10	38.27985	-76.9464	0.00	0.00	408.10	0.00	2.93	6.30	0.09	51.90	102.04	21.85	2.52	0.00	7.99
11	38.27308	-76.9533	0.00	35.29	397.58	0.00	1.35	12.03	0.00	63.19	102.12	12.29	1.68	5.41	5.28
12	38.27505	-76.9311	0.00	19.05	417.53	3.27	7.21	8.82	0.13	61.59	106.08	13.23	2.12	39.31	6.49
13	38.26944	-76.9385	0.00	18.83	414.04	1.50	0.00	9.51	0.00	62.49	106.12	12.20	6.48	0.00	4.50
14	38.26617	-76.9464	0.00	3.51	411.38	0.00	2.45	7.09	0.11	61.43	104.88	14.22	1.14	1.38	6.58
15	38.27277	-76.917	0.02	0.00	570.74	1.18	0.00	5.01	0.06	54.18	126.01	7.08	1.32	1.10	3.97
16	38.26244	-76.924	0.00	15.10	423.52	1.57	1.20	7.40	0.09	58.22	108.17	14.88	14.37	18.15	7.27
17	38.25542	-76.9419	0.01	0.00	592.24	1.27	0.00	5.86	0.01	54.67	130.28	10.41	4.89	5.91	3.48
18	38.23385	-76.9104	0.11	6.30	634.58	1.08	0.00	4.76	0.22	60.03	140.91	12.08	4.88	1.01	3.81
19	38.25155	-76.8657	0.10	0.00	694.80	1.63	0.00	3.09	0.02	70.39	153.30	15.07	5.83	4.02	4.54
20	38.22765	-76.8805	0.08	0.00	617.65	0.00	1.18	3.83	0.00	50.80	136.42	14.20	3.13	1.53	8.64
21	38.20565	-76.8896	0.04	4.23	622.10	0.00	0.00	3.32	0.00	55.08	137.73	8.34	1.87	0.00	8.69

Near-Bed Water Trace Metal Data (ICP-OES)

Site	Latitude	Longitude	Al [ppm]	As [ppb]	B [ppb]	Cd [ppb]	Cr [ppb]	Cu [ppb]	Fe [ppm]	Li [ppb]	Mg [ppm]	Mn [ppb]	Ni [ppb]	Pb [ppb]	Zn [ppb]
1	38.32762	-77.0197	lost during transport												
2	38.33036	-76.9997	0.78	3.74	536.54	0.00	0.00	26.33	1.83	35.61	113.71	193.72	4.55	10.88	18.87
3	38.31157	-77.0111	0.09	3.98	584.61	0.00	0.00	5.41	0.07	38.97	135.37	5.42	3.07	4.99	9.68
4	38.31897	-76.9702	0.07	13.23	684.52	0.00	0.00	1.65	0.00	51.64	148.86	10.99	1.46	3.24	4.67
5	38.29606	-76.9872	0.09	2.78	755.90	0.00	0.00	2.27	0.04	60.38	164.53	242.27	1.82	0.00	3.15
6	38.29129	-76.9618	0.13	7.70	430.12	0.00	2.07	8.06	0.59	62.21	109.52	64.00	1.41	0.00	13.08
7	38.2932	-76.9458	0.08	0.00	782.49	1.43	1.16	0.00	0.00	60.99	170.80	151.98	6.68	1.51	8.94
8	38.27543	-76.964	0.22	10.10	847.24	1.54	0.00	3.35	0.07	94.41	182.99	27.49	2.15	0.00	5.11
9	38.2826	-76.9436	1.99	16.18	589.08	3.41	8.43	15.71	8.26	87.84	144.28	913.30	16.01	1.93	64.43
10	38.27985	-76.9464	4.22	16.10	469.87	1.17	14.92	19.63	11.29	72.47	118.11	582.29	25.47	0.00	87.04
11	38.27308	-76.9533	0.07	10.82	450.65	2.58	4.18	7.89	0.64	62.00	114.54	62.51	4.32	0.00	22.67
12	38.27505	-76.9311	0.37	17.89	610.58	2.11	3.33	12.61	1.16	92.38	149.48	145.65	6.46	0.00	18.95
13	38.26944	-76.9385	6.71	28.47	496.53	2.16	29.90	41.65	25.95	81.47	119.42	1543.42	32.65	49.63	209.51
14	38.26617	-76.9464	1.83	6.05	456.89	3.32	8.78	16.85	7.99	71.70	111.76	943.48	20.11	0.00	72.24
15	38.27277	-76.917	0.15	0.00	803.37	0.00	1.20	2.38	0.16	60.10	169.99	17.77	3.84	1.18	5.32
16	38.26244	-76.924	2.22	7.15	472.30	2.12	10.73	16.78	8.34	72.09	130.88	488.68	2.62	33.77	83.07
17	38.25542	-76.9419	0.13	0.00	828.94	1.79	0.00	0.00	0.10	67.05	175.86	1072.63	0.00	2.20	5.16
18	38.23385	-76.9104	0.39	4.38	928.57	1.48	0.00	5.42	0.57	66.30	197.03	36.90	1.28	0.00	8.44
19	38.25155	-76.8657	0.05	2.56	774.67	0.00	0.00	2.06	0.00	52.15	168.48	25.39	5.12	2.17	3.83
20	38.22765	-76.8805	0.08	3.12	986.79	0.00	0.00	0.00	0.09	75.33	207.23	17.38	0.00	0.00	3.92
21	38.20565	-76.8896	0.10	3.28	895.41	1.12	0.00	5.67	0.07	66.19	189.42	13.60	2.23	1.76	8.93

Sediment Trace Metal Data (ICP-OES)

Site	Latitude	Longitude	Al (ppm)	As (ppm)	B (ppm)	Cd (ppm)	Cr (ppm)	Cu (ppm)	Fe (ppm)	Li (ppm)	Mg (ppm)	Mn (ppm)	Ni (ppm)	Pb (ppm)	Zn (ppm)
1	38.32762	-77.0197	4908.00	6.48	21.31	0.92	23.91	18.57	18595.00	45.69	2379.00	572.50	16.46	9.44	76.45
2	38.33036	-76.9997	7566.00	5.95	50.52	0.20	48.25	20.95	24705.00	18.42	3952.00	1246.50	26.10	24.29	121.35
3	38.31157	-77.0111	9353.00	6.11	54.76	0.12	67.03	22.55	26687.00	24.09	4407.00	1676.90	39.16	26.20	134.81
4	38.31897	-76.9702	10095.00	9.69	54.34	0.21	54.02	19.58	28151.00	29.77	4045.00	570.10	29.40	23.81	113.08
5	38.29606	-76.9872	8693.00	6.10	47.53	0.21	49.04	21.52	25959.00	23.53	4145.00	748.40	29.67	25.11	136.48
6	38.29129	-76.9618	9978.00	12.54	33.73	0.87	46.30	216.98	32045.00	49.96	4470.00	1048.80	30.45	28.74	143.38
7	38.2932	-76.9458	7985.00	10.37	44.10	0.37	44.58	19.61	26310.00	25.11	3530.00	409.90	27.07	25.54	130.91
8	38.27543	-76.964	6212.00	5.11	34.66	0.17	35.69	18.82	20014.00	18.99	3087.00	481.10	24.89	23.12	119.94
9	38.2826	-76.9436	9027.00	5.38	35.94	2.41	36.59	55.62	27236.00	48.31	4963.00	744.30	26.86	22.61	128.04
10	38.27985	-76.9464	9686.00	11.23	36.54	0.95	38.22	46.62	29175.00	56.45	4914.00	718.50	26.60	32.87	143.58
11	38.27308	-76.9533	10457.00	9.29	36.27	0.66	40.52	43.72	29438.00	55.40	5039.00	819.20	28.71	28.09	138.36
12	38.27505	-76.9311	9605.00	10.19	36.97	0.70	37.84	28.44	30908.00	51.53	4349.00	601.30	26.44	19.68	123.96
13	38.26944	-76.9385	9233.00	10.66	35.35	0.87	35.79	30.87	27719.00	36.00	5158.00	668.20	26.01	21.08	132.78
14	38.26617	-76.9464	6970.00	6.23	28.74	0.81	29.86	26.33	22927.00	37.76	3918.00	579.80	22.15	18.03	108.99

15	38.27277	-76.917	3821.00	6.43	25.97	0.33	23.56	14.12	15137.00	14.52	2023.00	200.80	17.74	16.75	102.00
16	38.26244	-76.924	9711.00	7.54	37.40	1.11	39.29	38.45	28963.00	50.95	5440.00	674.30	26.99	17.54	136.86
17	38.25542	-76.9419	4090.00	4.13	25.52	0.18	23.53	14.32	14098.00	13.96	2207.00	260.00	17.96	17.40	96.21
18	38.23385	-76.9104	4910.00	7.26	29.19	0.38	27.10	21.40	18043.00	21.50	2788.00	274.10	25.24	27.26	146.87
19	38.25155	-76.8657	5606.00	7.44	30.24	0.41	28.58	20.65	17613.00	23.88	2825.00	204.20	24.74	25.76	133.42
20	38.22765	-76.8805	4740.00	6.13	28.68	0.37	25.94	23.69	16862.00	20.40	2740.00	241.80	23.51	26.16	137.76
21	38.20565	-76.8896	4766.00	7.14	30.15	0.42	27.56	18.30	17480.00	18.28	2789.00	237.60	22.60	24.22	124.55
